# Engineering vertically interrogated interferometric sensors for optical label-free biosensing

**DOI:** 10.1007/s00216-020-02411-3

**Published:** 2020-02-14

**Authors:** Rafael Casquel, Miguel Holgado, María F. Laguna, Ana L. Hernández, Beatriz Santamaría, Álvaro Lavín, Luca Tramarin, Pedro Herreros

**Affiliations:** 1 Applied Physics and Materials Engineering Department, Escuela Técnica Superior de Ingenieros Industriales, Universidad Politécnica de Madrid, C/ José Gutierrez Abascal, 2, 28006 Madrid, Spain; 2 Optics, Photonics and Biophotonics Group, Centre for Biomedical Technology, Campus de Montegancedo Universidad Politécnica de Madrid, 28223 Pozuelo de Alarcón, Madrid, Spain; 3 Mech, Chem & Industrial Design Engineering Department, Escuela Técnica Superior de Ingenería y Diseño Industrial, Universidad Politécnica de Madrid, Ronda de Valencia 3, 28012 Madrid, Spain

**Keywords:** Photonic calculations, Optical sensors, Interferometric sensors, Nanofabrication, Biosensing

## Abstract

In this work, we review the technology of vertically interrogated optical biosensors from the point of view of engineering. Vertical sensors present several advantages in the fabrication processes and in the light coupling systems, compared with other interferometric sensors. Four different interrelated aspects of the design are identified and described: sensing cell design, optical techniques used in the interrogation, fabrication processes, fluidics, and biofunctionalization of the sensing surface. The designer of a vertical sensor should decide carefully which solution to adopt on each aspect prior to finally integrating all the components in a single platform. Complexity, cost, and reliability of this platform will be determined by the decisions taken on each of the design process. We focus on the research and experience acquired by our group during last years in the field of optical biosensors.

## Introduction

Biosensing devices based on optical devices have had an important development during past decades. First and probably most important sensor from both academic and commercial point of view are the devices based on surface plasmon resonance, firstly reported in 1983 [1] and extensively studied during following years [2, 3]. Guided wave optical sensors [4] based on planar devices emerged due to the improvement of nanofabrication techniques for photonics; they usually consist of interferometers, where the position of the optical mode is tracked as a function of the concentration of the bioanalyte to detect, or the refractive index of the liquid sample. Some examples are based on ring resonators [5–7], photonic crystals [8, 9], Young interferometer [10], Mach-Zenhder interferometers [11, 12] or bimodal [13] and trimodal [14] waveguides, and grating couplers [15], among other typologies of devices. Several reviews cover extensively optical label-free biosensors, comparing also values of sensitivity and limit of detection for a variety of devices [16, 17], emerging technologies [18], or advances in silicon-based optical biosensors [19].

Instead of planar devices, the use of vertically interrogated interferometric devices may imply less complex optical coupling, and can also have advantages from the fluidic and biofunctionalization point of view. In this work, we review how to model from the point of view of engineering optical sensors based on vertical cavities in order to not only maximize sensitivity but also integrate all optical components and obtain a reliable and feasible device. Our research group has been working on this field during last decade, developing new typologies of devices, mainly based in vertical sensing cells, from the point of view of engineering devices.

## Materials and methods

### Vertical sensors

We define vertical sensors as optical devices interrogated at normal incidence, using generally a non-complex optical setup, with a light source, a focusing system and a light collector, which can measure intensity (photodetector) or analyse the reflected or transmitted light (spectrometer, CCD camera). This gives a clear advantage from planar devices, in which the coupling of the light from fibre to waveguide requires an important effort, using systems such as grating couplers and inverted tapers to have an efficient coupling [20]. Another advantage is that the fabrication processes involved are generally less complex compared with waveguide-based sensor, which require high accuracy of fabrication; otherwise, optical losses and uncertainty of the measurement are too high.

The simplest vertical interrogated photonic device is a Fabry-Perot resonator consisting of a single dielectric layer fabricated on a substrate. This substrate can be either transparent or absorbent depending on whether the optical signal is measured by reflectance or by transmittance. The upper layer is biofunctionalized and there is a shift in the optical signal when the biorecognition takes place. Several works are examples of this typology of device [21].

Structuring the surface of the layer by means of the fabrication of pillars [22–24], holes [25–27], or using a layer of porous material could increase the sensing surface of the sensing cell, besides presenting a particular confinement of the light [28] which may result in a better sensitivity or limit of detection. Biosensors based on porous silicon were reported in 1997 [29] and many variations on the same biosensing principle have been also reported since [30], using layers with a variety of porosity values and configurations. Porous layers can be replaced for other typologies of nanostructures, with similar results, such as ZnO nanostructures [31], with high versatility of shapes and sizes.

**Fig. 1:**
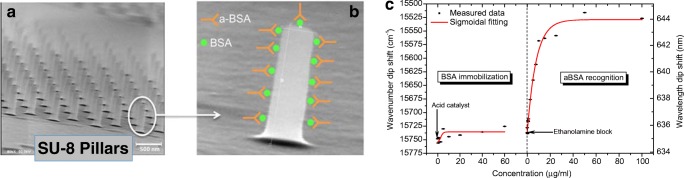
Nanometric SU-8 pillars vertically characterized. From [32]. **a** SEM caption of an array of pillars. **b** Functionalization of BSA and recognition of anti-BSA on an single pillar. **c** biorecognition curve

In 2010, we reported a photonic sensor called BICELL (Biophotonic sensing cell) based on SU-8 nanopillar arrays, characterized vertically by reflection [32] (with silicon substrate) and by transmittance (with transparent substrate) [33]. The use of nanopillars increased the sensing area and improved the limit of detection, reaching an anti-BSA limit of detection of 2.3 ng/mL, an anti-gestrinone limit of detection lower than 1 ng/mL [33]; it also showed good results in the biorecognition of dry eye disease biomarkers [34] and for dengue immunoassay [35]. Besides, theoretical calculations presented good correlation with experimental results. Figure 1 shows a SEM caption of the array of SU-8 pillars over SiO_2_ and a biorecognition curve of anti-BSA using arrays of SU-8 nanopillars as sensing cell.

A step forward of using a single-layer Fabry-Perot interferometer is the vertical configuration of a microcavity by means of two Bragg reflectors composed of a pair of materials. The aim of using such a cavity is reducing the width of the optical resonance, which may improve the uncertainty of the detection. Besides, there should be space in the Bragg reflectors and the cavity to be fulfilled with fluid or biosample for the detection. This can be done clearly with porous materials, for example with porous silicon controlling the size of the pore, matching the values of the refractive index to build a working Bragg reflector, reaching a limit of detection in the order of 10^−7^ RIU [36], or with a similar approach using porous SiO_2_ and TiO_2_ layers [37].

**Fig. 2:**
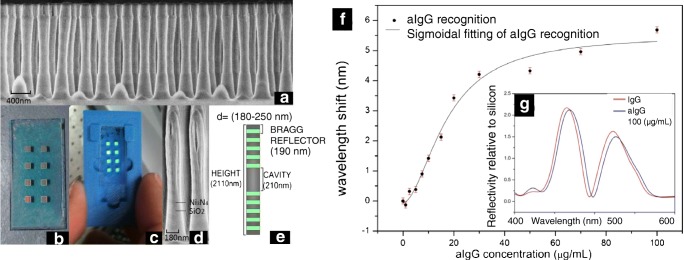
Left: Schematic of resonant nanopillars arrays. Optical response of resonant nanopillars. **a** SEM caption of fabricated R-NPs. **b** Chip of eight sensing cells. **c** Fluidic holder. **d** SEM detail of a pillar. **e** Dimensions and layers of the pillar. [Reprinted/Adapted] with permission from [43] © The Optical Society. Right: **f** Biorecognition curve of anti-IgGs from a R-NPs cell. **g** Reflectivity as a function of wavelength for a R-NPs cell, saturated with IgGs and after anti-IgGs recognition. [Reprinted/Adapted] with permission from [44] © The Optical Society

One of the main disadvantages of porous materials for sensing is that, whereas the infiltration of fluid is not complex and the results in refractive index sensing are excellent due to the high sensing space available, for biosensing, the cleaning process could present problems, with undesired material in the sensing area. An evolution from the SU-8 BICELLs, whose cleaning process did not present problems, and also acting as a microcavity are the resonant nanopillars arrays (R-NPs) [38]. These sensing cells were fabricated from a bulk multilayer stack over transparent substrate, consisting of two Bragg reflectors of Si_3_N_4_/SiO_2_ and a central cavity of SiO_2_. The process of fabrication included a lithography step, which could be either laser interference lithography [39] or E-beam lithography [40], obtaining pillars with around 200 nm in diameter, several values of pitch and a height in the order of 2 μm. (Fig. 2, left).

Previous single microresonator pillars have demonstrated high values of Quality Factor (Q-Factor) [41] with different semiconductor materials [42]. In the R-NPs, the optical response of the pillars presents a photonic band gap and a centred resonance, with a not as high Q-Factor resonance as other resonators, but much better compared with single-layer F-P cell. The use of resonant nanopillars represents certain advantages from other devices, not only because of the shape of the resonance compared with a monolayer Fabry-Perot, but also since the cleaning of the device is not complex. This typology of device has been developed by our group for biochemical sensing [43] and recognition of anti-IgGs [44], reaching a refractive index sensitivity in the order of 400 nm/RIU or higher, and a detection limit of anti-IgGs in the order of ng/mL (Fig. 2. The sensor can be used either in continuous mode or in static mode. This will be analysed at the end of this work, since represents advantages from the sensing point of view given several conditions.

### Engineering vertical sensors

**Fig. 3:**
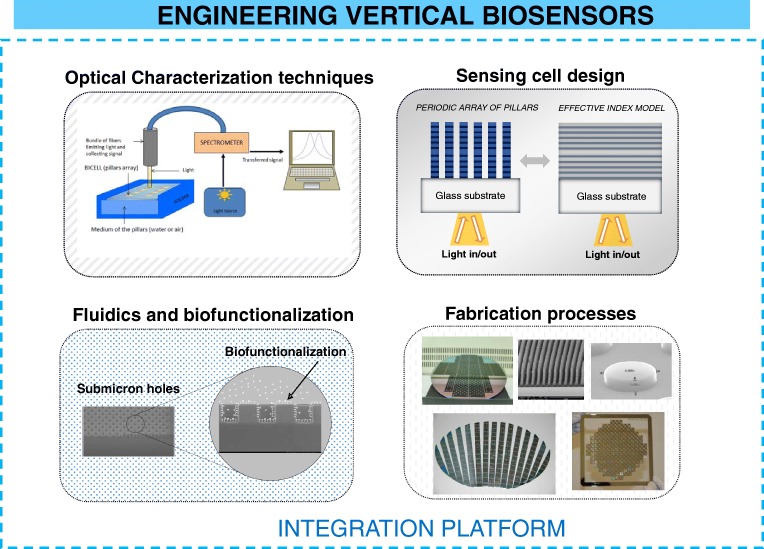
Areas of interest of vertical Interferometric biosensors to be integrated on a single platform

In terms of engineering, we have to focus on all the relevant areas for the biosensor, and not only in improving a particular figure of merit. This task is quite challenging, but as a result can provide a feasible biosensor and with potentially commercial applications. We have divided all the aspects of the biosensor into four sections: design of the geometry of the sensing cell, optical techniques used in the interrogation, fabrication processes, fluidics, and biofunctionalization of the sensing surface. Finally, we have to consider that all the parts must be integrated in a single platform. All these aspects are summarized in Fig. 3. The complexity of the integration finally will determine an important part of the total cost of the device. During the next subsections, we will discuss each of these areas.

### Design of the geometry of the sensing cell

Before designing the biosensor, we have to consider how to calculate the sensitivity and limit of detection of the sensor. The sensitivity of an optical sensor is well defined by the relation of the shift of the optical mode and the change in the refractive index of the fluid, for refractive index sensing, and the relation between the shift and the increase of the analyte concentration (linear range) for biosensing. In terms of refractive index sensitivity, typical values are in between 100 nm/RIU and 1000 nm/RIU [45]. Values of refractive index limit of detection range from 10^−4^ to 10^−7^ RIU, whereas limit of detection for biosensing depends on the affinity of the antigen-antibody reaction; typical values are in the range of nm/mL for most immunoassays.

Refractive index sensitivity is often used to compare performance of optical biosensors; however, we have to be careful with this comparison because limit of detection also takes into account the uncertainty of the measurement. The work of Lavín [46] proposed a generalized method to estimate the limit of detection of a biosensor focused mainly in the uncertainty calculation, where this uncertainty not only depends on the resolution of the optical tool used (i.e. the resolution of the spectrometer) but also with the shape and characteristics of the optical mode. Higher Q-factor resonance and signal to noise ratio signals result in a lower value of the statistical standard deviation of the measurement, and finally in a lower uncertainty. White and Fan [45] stated that there is a linear relation of the standard deviation of the measurements with the optical shift and an exponential relation with the signal to noise ratio. A sensor with a low refractive index sensitivity (25 nm/RIU) had the same limit of detection than other with higher sensitivity (1000 nm/RIU), but with lower Q-Factor (10^4^ compared with 10^7^).

For the calculation of the optical response of the sensor, we have available optical software as Rsoft [47], with its module Fullwave [48], which calculates reflectivity of the light on a complex surface such as a nanopillar patterned array, using 3D finite difference time domain method for calculating Maxwell equations under no internal charges condition. With this method, results are usually quite accurate; its disadvantage is that is time-consuming, and presents some problems when considering spatial grids in the order of nanometres. This is of high importance when simulating a layer of antibody-antigen pair, with a thickness in the order of several nanometres when the reaction starts to saturate, and of 1 nm or lower for small concentrations of analyte biorecognized.

**Fig. 4:**
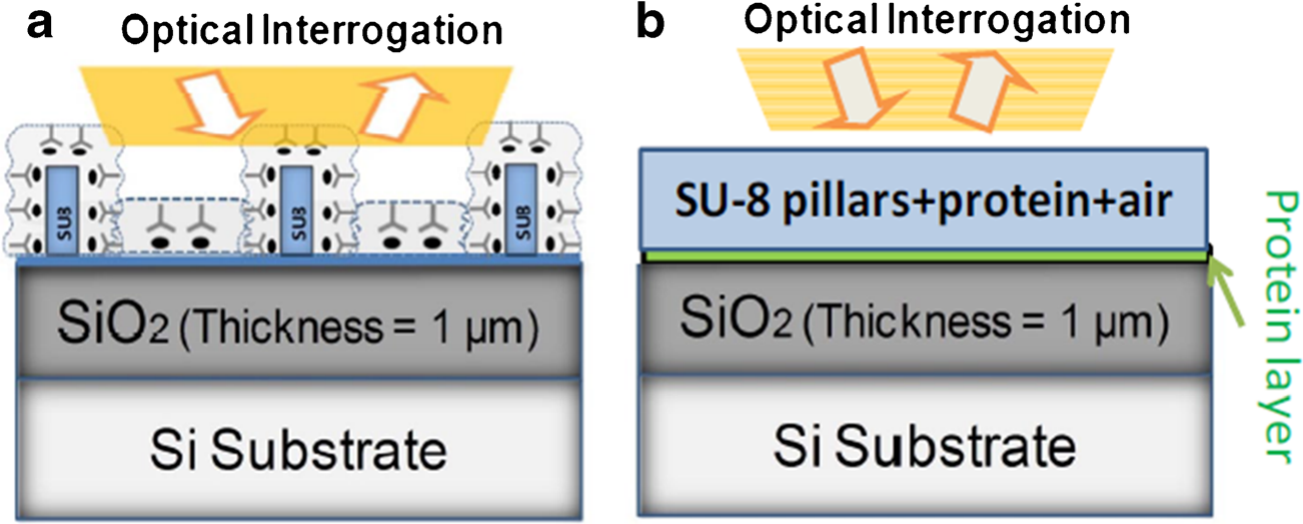
**a** Schematic of the layout of characterized Su-8 nanopillars. **b** Equivalent thin film model used for analytical calculations. From [49]

Another way to proceed in the design of the sensor is using an analytical method to calculate the optical response. The calculation of the optical response of a multilayer can be done with any Mathematical software. The layer consisting of pillars can be calculated as an equivalent thin film with an effective refractive index. An example of this is shown in Fig. 4. The SU-8 pillars are calculated as an equivalent layer consisting of a mixture of SU-8, air, and a biolayer of several nanometres.

Using such a simplified model, the optimization of the geometrical parameters of the biosensor, which includes diameter of the pillars, height, and pitch, is immediate. For example, considering a constant diameter of 200 nm, and a height of 750 nm, the optimal value found for the pitch is 400 nm, with an expected shift in the optical mode four times higher than in previous work [49]. With this model, we can also evaluate not only the total shift of the resonance, but also the Q-factor of the measured resonance and its throughput, for a wide variety of geometrical configurations, with an automated calculation method [50].

**Fig. 5:**
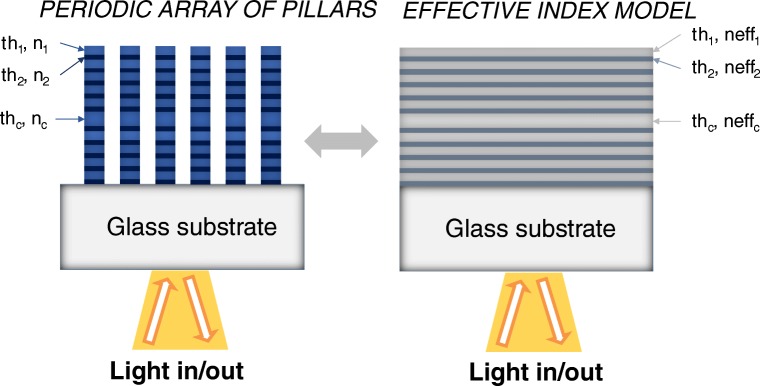
Equivalent 1-D model for resonant nanopillars

We can extend the 1-D equivalent model to multilayer structures, such as Fabry-Perot resonators consisting of Bragg reflector porous material based, or resonant nanopillars, using an equivalent refractive index for each layer (Fig. 5). This refractive index can be calculated using effective medium theory [51]. This theory has been used extensively to calculate optical properties in porous materials, on a single-layer or multilayer structures [36], and can also be used in nanopatterned materials [52].

Effective index can be calculated using different effective medium models: Maxwell Garnett, Brueggeman, or volumetric method. Recent simulations have shown better results for volumetric method compared with Maxwell Garnett and Brueggeman. Using this model for optimization allows not only calculating optimal parameters for values of diameter and pitch for the pillars but also considering different materials for the multilayer material used as base substrate. Preliminary results using these calculation techniques show that the shift of the resonance as a function of refractive index of the surrounding fluid is similar to the experimental values. However, there is a shift in the calculated position of the resonance (around 20 nm) compared with experimental values, probably because the effective medium models does not cover completely the effect of confinement into the pillars, and thus the results can be used to analyse trends and to compare sensitivity between sensors, but need to be supported by 3-D FDTD calculations.

### Fabrication of the sensing cell

The fabrication process of the sensing cell represents probably the most important cost in economic terms of all the processes involved in obtaining the biosensor, in particular when processes of e-beam lithography are involved. This is the main advantage of using a single-layer Fabry-Perot resonator as sensing cell, and porous material microcavities.

Fabrication of a single-layer F-P interferometer can be done with thin film growth techniques, such as plasma-enhanced chemical vapour deposition (PECVD) or magnetron sputtering, depending on the materials and the optical properties required, or with an spin coater in case the material is a polymer such as SU-8. Porous silicon layers are typically fabricated with two techniques: electrochemical etching and strain etching. Similar layers as columnar nanostructures of SiO_2_ and TiO_2_ [52] are fabricated using physical vapour oblique angle deposition (PVOAD).

Arrays of R-NPs have been fabricated using e-beam lithography and inductively plasma coupling etching [40], with laser interference lithography and ICP etching, a process described elsewhere [39]. The maximum height reached was in the order of 2 μm, with diameters between 200 and 300 nm and a minimum value of pitch of 400 nm. The fabrication with laser interference lithography [53] has the advantage that a large area of mask photoresin can be exposed at the same time using the interference between two or more beams, whereas the minimum size of pillars obtained with e-beam is lower than with LIL. Other technique that could be used for the fabrication of pillars and holes [54] at nanometric level is deep UV lithography, with light sources in the order of 248 nm or lower.

Nanopillars and resonant nanopillars could be also fabricated using nanoimprinting lithography (NIL); this is interesting for reducing the unitary cost of each sensing cell. Some examples of fabrication with NIL are silicon nanopillars in the order of 40 nm with a height of 3 μm [55]. The advantage of NIL is that the lithography is made by one single step and could represent a much lower cost when a high number of sensing cells are fabricated on a single wafer.

One advantage of vertical sensors is that the fabrication process is not as critical as in other optical sensors, for example in ring resonators, where imperfections in the process results in high scattering losses, and a reduction on the limit of detection of the device. On vertical sensing cells, a higher sensing area is generally characterized, for example in R-NPs, we used sensing cells of 1 × 1 mm or 500 × 500 μm; small defects on the fabrication of the arrays have slight influence in the final sensitivity and limit of detection of the biosensor. For porous layers, where it is not easy to reach a high reproducibility of fabrication processes, a variation will represent a small shift in the optical resonance that in most cases will not affect to the performance of the sensor.

### Optical techniques

Regarding optical techniques, for interferometric vertical sensors spectrometry is by far the most widely used technique. With spectrometry, we characterize the sensing area whose size is determined by the beam spot, this spot could cover from several microns to millimetres. Lower sensing areas are interesting for having highly integrated sensing cells, and for using low volumes of target sample for the detection. The range of the light covered with spectrometry is variable, but usually ranges from visible light to near infrared are chosen (400–1100 nm). The instrument can use a diffraction grating to separate into wavelengths, or a more complex system, such as a Michelson interferometer, with a Fourier transform operation to calculate the spectrum. The signal to noise ratio and the wavelength resolution of the instrument will have influence in the calculation of the uncertainty of the measurement. Spectrometers can be also composed of several measurement channels; this is useful for multiplexing sensing, for example of 16 channels measuring arrays of R-NPs [56].

**Fig. 6:**
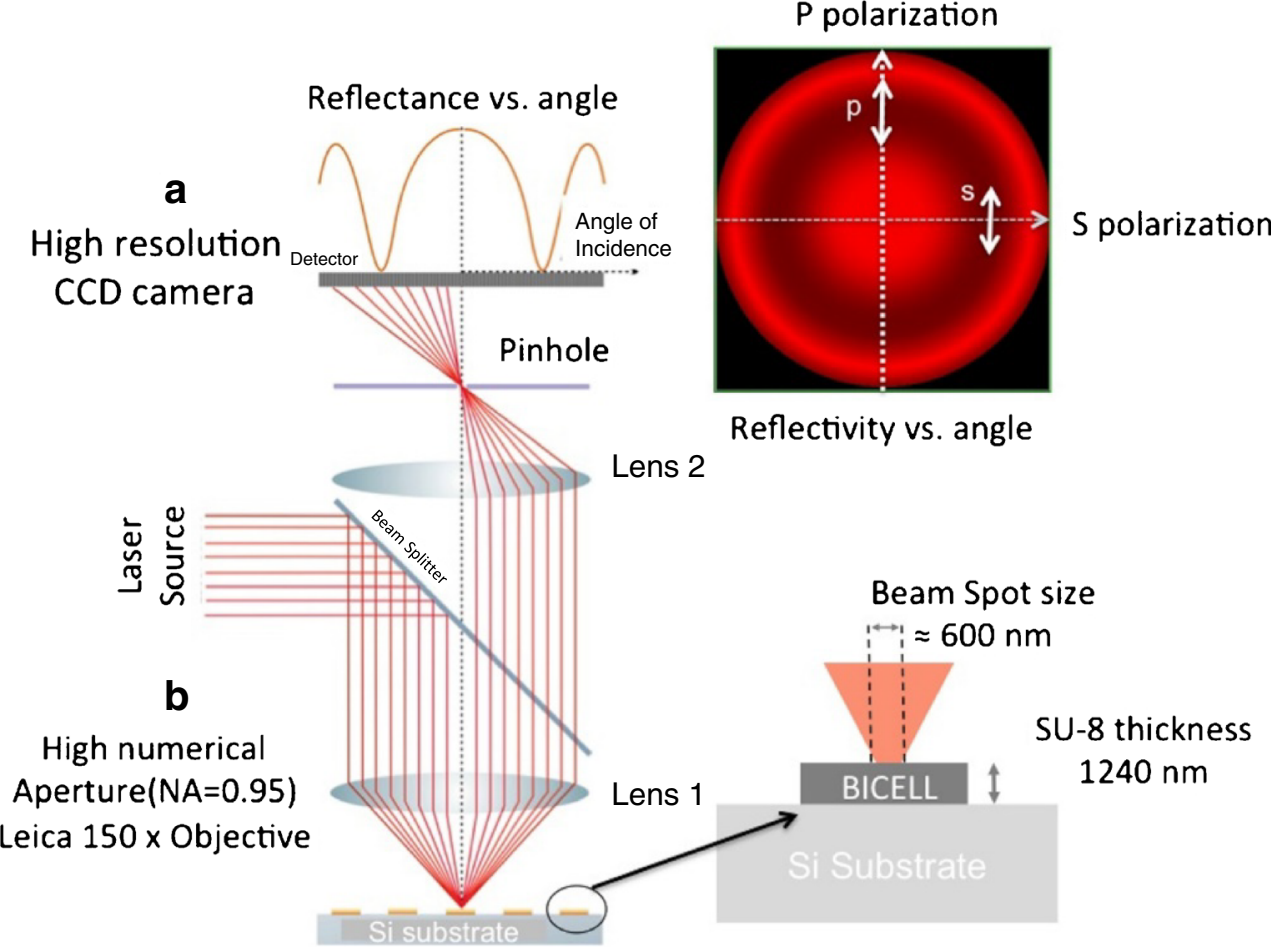
Schematic of reflectometry at profile level. The optical setup focuses the laser beam on the surface of a sensing cell with a sub-micrometric spot. The system obtains reflectivity for a variety of angles, for pure polarizations s and p, and for a combination of polarization states. [Reprinted/Adapted] with permission from [59] © The Optical Society

By using monochromatic, and in particular laser sources, lower spot sizes can be reached. Beam profile reflectometry [57] and beam profile ellipsometry [58] are techniques used in metrology of dielectric thin films, and are useful for measuring the reflectivity of light as a function of the angle of incidence and the change in polarization state in sub-micrometric domains, due to the spot size reached (0.9 μm for a 675 nm laser). By adapting these techniques, we can reach even a lower spot size with a microscope objective of × 150, these are the so-called reflectometry and ellipsometry at profile level (RPL and EPL). These techniques allow characterizing the sensing surface with a sub-micrometric spot [59]. The system uses a laser source emitting at 637 nm with a high numerical aperture microscope objective (0.95). The range of angles covered is from − 72 to 72 degrees, and also measures the reflectivity for both polarization states (Fig. 6).

**Fig. 7:**
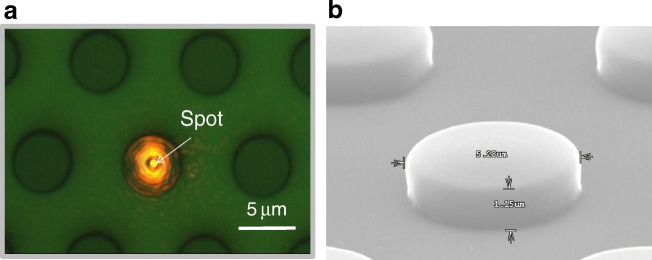
**a** Top view of a lattice of SU-8 pillars with a pith of 10 μm, and a diameter in the order of 5 μm, with the laser source of RPL focused on the centre of a pillar. **b** SEM caption of a single pillar with 1.15 μm in height and 5.28 μm in width

The possibility of using a sub-micrometric spot allows localized label-free biosensing in small areas, such as sub-micrometric holes [25] or pillars. We used these techniques for biorecognizing anti-BSA over circular area of SU-8 over silicon functionalized with BSA with a limit of detection comparable with high resolution spectrometry [59]. The characterization of such small sensing areas can be interesting for increasing the integration of the sensing cell, or to measure variations of biofunctionalization or recognition on smaller areas. Figure 7 shows the laser spot focusing on a micropillar of 5 μm of diameter. The system could be able to characterize solely the reflectivity of a single pillar, and then control the biofunctionalization and biorecognition on its surface.

Measuring the intensity of the reflected signal instead the shift of an optical mode can strongly simplify the optical setup, since no spectrometer or angle-resolved camera is needed. This concept, using the technology of compact discs, DVD, and Blu-Ray has been developed for several immunoassays [60, 61], reaching competitive values of limit of detection. The light source characterizes in this case circular spots in the order of 100 μm, and besides reducing complexity of optical setup, the system is completely standardized, also from the point of view of positioning. The authors have characterized arrays of 10 × 10 spots with the automated position system; this can be also used for multiplexing.

The combination of the measurement of the optical intensity in a given spectral range with the interferometry in two interferometric sensing cells is a method to enhance the biosensing system as a whole. This interferometric optical detection method (IODM) has been detailed elsewhere [62–64], has been patented [65], and is under commercial exploitation by bio optical detection (BioD) [66].

**Fig. 8:**
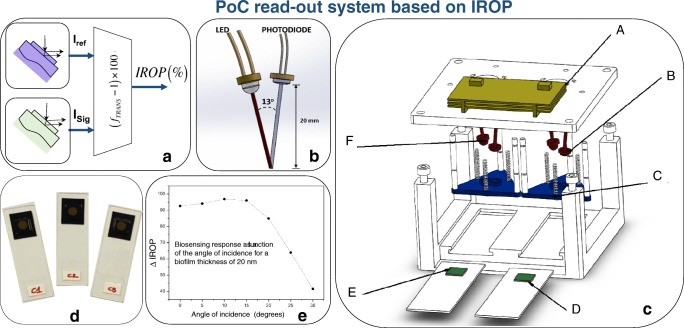
PoC readout system based on IROP. **a** Block diagram of the functionality of the PoC: IRef generates the interferometric reference and ISig produces the interferometric signal as a result of the biological accumulation. fTRANS is the operation between both signals and delivers IROP (%). **b** Optical layout of the LED and photodiode. **c** Hardware components. **d** A set of KITs based on FPIs as biosensors. **e** Biosensing response as a function of the angle of incidence for a biofilm thickness of 20 nm. From [64]

Several implementations can be carried out, but the one in which the interference of two interferometers is expressed as a variation of the optical power is relevant for the envelopment of reliable point of care and point of need devices (see [64]). In this case, a reference interferometer is interrogated in a given spectral range and compared with a signal interferometer (the one exposed to the accumulation of recognition of biological material). The interference response changes due to this accumulation of specific recognition of biological material. As a result, this interferometric change provokes a change in the intensity, which compared with the intensity of the reference interferometer give us the readout signal expressed in increased relative optical power (IROP). Figure 8 shows the schematic of a point of care system based on IROP concept, and its main components.

### Fluidics and biofunctionalization of vertical biosensors: influence of the environment of measurement conditions

The biofunctionalization of the sensing cells in optical biosensors is a key process to obtain a competitive device, since it is one of the main components of the biosensor. In this sense, a good specificity and sensitivity to the analyte of interest is a key point in the development of a biosensor. In the surface biofunctionalization, factors like an optimal density of functional groups, favourable orientation, good accessibility to the target, low non-specific binding, and stable linkage between the bioreceptor and the surface are essential for a final biosensor performance.

There are different surface modification strategies for optical biosensors functionalization according to the sensing surface and the bioapplication [67, 68]. To summarize, there are four immobilization strategies: physical adsorption, chemical technique by self-assembled monolayer (SAM), affinity, and entrapping in polymers. The silicon technology is preferred in optical biosensors and the surface chemistry for bioreceptor attachment on silicon-based materials has been widely studied [69].

For chemical surface modification by self-assembled silane, organofunctional alkoxysilanes are used. The reaction over the sensing surface is based on the condensation between the siloxanes of the organosilane and hydroxyl groups present on the surface. This chemical modification is more complex than thiol chemistry over gold surfaces. There is a great variety of commercial organosilanes. The -NH_2_, -SH, -COOH, epoxy functionalities are mainly used [70].

**Fig. 9:**
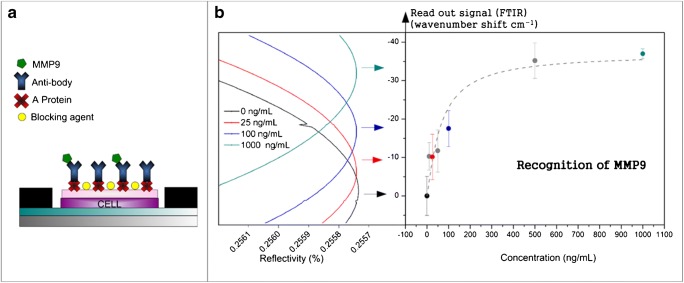
**a** Sensing surface biofunctionalized with A-protein and anti-MMP9. **b** Biorecognition of MMP9. From [71]

Within the research work of our group in vertical biosensors, we have functionalized different sensing cells, as cells based on F-P sensors, SU-8 nanopillars, and multilayer resonant nanopillars, among others. An example of F-P sensors is shown in Fig. 9. It is based on a planar cell of two interferometric layers of SiO_2_ and SU-8, plus a thin layer of nitrocellulose with around 75 nm in thickness. This upper layer facilitates the electrostatic biofunctionalization of A-protein linker in order to obtain a better orientation of the antibodies. We have demonstrated the detection of MMP9 [71] protein related to dry eye disease and the detection of C-protein related to inflammatory, cardiovascular, and infectious processes.

An example of nanostructured materials is the SU-8 nanopillar arrays sensor [32]. The SU-8 is an epoxy-based negative photoresist that can be nanostructured by exposure to UV. By immersion of the sensing cells in sulphuric acid 95%, the SU-8 epoxy groups are open and it is possible to immobilize bioreceptors such proteins by covalent attachment. This material is of great interest because it is easy to increase the response of biosensor by nanostructure fabrication and the attachment of bioreceptor is performed via amine group without an intermediate linker. With this typology of biosensor, we demonstrated the biodetection of anti-BSA [32] and anti-gestrinone [33, 72].

**Fig. 10:**
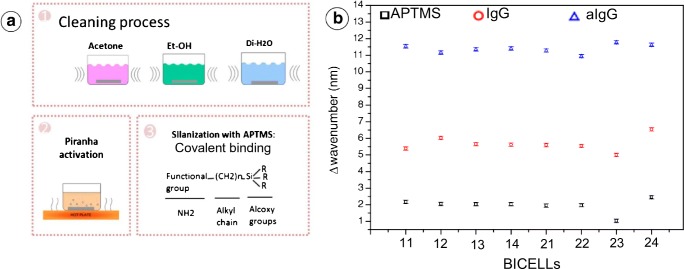
**a** Silanization of R-NPs. **b** Comparison of the mode shift from the silanization and anti-IgGs recognition for eight arrays of a single chip. [Reprinted/Adapted] with permission from [44] © The Optical Society

As previously mentioned, silanization process in nanostructured substrates is a crucial task. In the R-NPs-based sensor formed by SiO_2_ and Si_3_Ni_4_ reported before [44], the silanization of the pillar sensing surface was performed by 3-aminopropyltrimethoxysilane for immobilizing IgG antibodies acting as a bioreceptor (Fig. 10), with a previous plasma processing for increasing the hydrophilicity of the pillars. The biofunctionalization was performed and compared in eight different sensing cells, showing homogeneous results for both biofunctionalization of IgG and biorecognition of anti-IgG (Fig. 10b), which demonstrates the robustness of the sensing cells and the biofunctionalization protocol.

Biofunctionalization in vertical sensors does not present great differences compared with other typologies of biosensors, such as planar devices. The biofunctionalization protocols usually depend on the materials of the device and the bioanalyte to be functionalized. This is mainly the case in F-P sensing cells, where we can even use an intermediate layer such as nitrocellulose for ease the biofunctionalization of A-protein; however in nanostructured materials the deposition of such a thin layer is more complex and present difficulties to control the thickness. Thus, surface modification protocols based on organic chemistry need to be developed in order to modify functional groups of the surface. These protocols have the drawback of being longer and more complex than resist based biofunctionalization protocols. Other drawback is that the size of the bioanalyte to detect is limited by the space between pillars. For example, for arrays of pillars with 250 nm in diameter and 400 nm in pitch, which fabrication is feasible, the minimum space between pillars is of 150 nm. On the other hand, main advantage of nanostructured surface is that the sensing surface is significantly higher and these increase the number of available bioreceptors to recognize the analyte, which results in a better value of sensitivity of the detection.

**Fig. 11:**
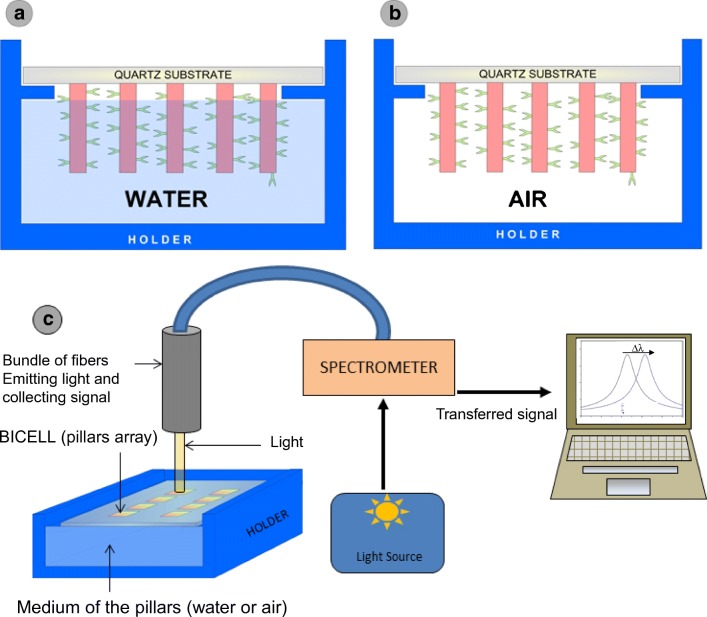
**a** Scheme of pillars immersed in water, **b** scheme of pillars in dry conditions, and **c** layout of the measurement system. From [73]

Finally, a not extensively studied aspect of the measurements is the environment of the sensing surface during the measurement [73]. The readout can be performed “on-line” with the sensing area immersed in fluid, typically PBS solution; in this case, the refractive index of the media is close to the value of the refractive index of water (Fig. 11a); the sensing cell can be also interrogated in the so-called dry conditions. On this occasion, the fluid is removed after the incubation process (Fig. 11b), and then the refractive index of the environment, which is considered to be air, is lower compared with wet conditions. The advantage of these measurements is that the contrast between the biolayer (which can be considered constant, with a value of 1.41 RIU) and the surrounding media is higher than in the case of PBS solutions.

**Fig. 12:**
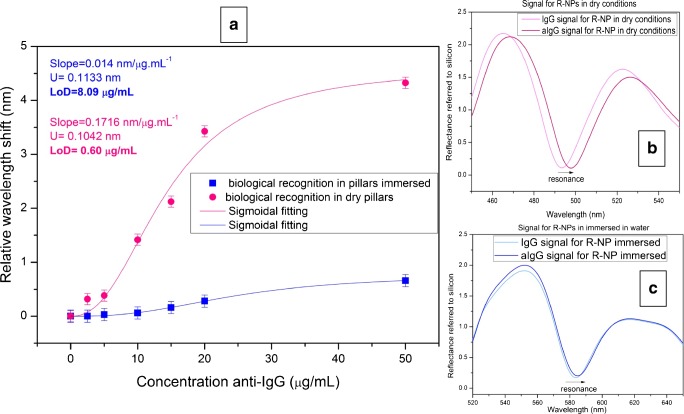
**a** Comparison of anti-IgG detection with wet and dry conditions. **b** Reflectivity of the sensing cell in dry conditions for IgG and anti-IgG saturation. **c** Reflectivity of the sensing cell immersed in water, for IgG and anti-IgG saturation. From [73]

Figure 12 shows the comparison between wet and dry conditions. The slope of the biorecognition is one order of magnitude greater in dry measurements compared with wet conditions. This experimental result is also supported with theoretical calculations, where the expected total shift of the immunoassay for dry conditions is clearly higher (12.7 nm compared with 2.31 nm).

The main disadvantage of this configuration of measurements could be the degradation of biomolecules after the removal of the fluid and exposition to the air. Being this possible for other bioapplications, we have not reported problems of this nature with IgG-anti-IgG, BSA-anti-BSA, and rotavirus detection. Other obvious disadvantage is that dry conditions do not allow on-line measurements, so there is not a possibility to study the kinetics of the recognition reaction; besides there is an extra cleaning process required for the measurement. When these two factors do not represent a problem for the measurements, dry condition is clearly a good option due to its higher performance. Furthermore, the fluidic system can be simplified since there is no need of microfluidic circuit to ensure the flow of the target fluid around the sensing surface. Usually simply dropping the fluid over the sensing surface or incubate during some time on a small vessel is enough to ensure the biofunctionalization and biodetection processes.

Figure 12 shows the comparison between the recognition curve of anti-IgGs using dry conditions and immersed in water, for the same R-NPs sensing cell.

The limit of detection reached is one order of magnitude lower for dry conditions, compared with the measurements in water (Fig. 12a). This is clear just by observation of the reflectivity spectra of both conditions, and comparing the shift of the anti-IgGs recognition (Fig. 12b, c).

### Discussion on pros and cons of vertical sensors: future perspectives

Main advantages and disadvantages of this typology of sensors are mentioned during the text, in the four aspects of the sensors studied. From the design of the sensing cell, the most important parameter to focus on is the sensitivity and limit of detection that can be achieved. Values of sensitivity in the order of several hundreds of nanometres per refractive index unit [43] or even 1000 nm/RIU [36] have been reported. These values are competitive with values of other sensors [45]. However, the values of Q-Factor of the optical mode of the interferometer are usually lower compared with other sensing cells, such as ring resonators, reaching Q-Factor values of 10^3^–10^4^ or higher [16], which results in a better value of limit of detection. This can be partially solved using resonant nanopillars or multilayer microcavities, in particular with materials of higher refractive index. Having a 1-D simplified model is also interesting for the designer of the sensor, because it allows not only the optimization of the device but also shorten the time of the optimization process.

Fabrication requirements of vertical sensing cells are generally lower compared with other devices. This is quite clear for monolayer F-P and porous materials, but also in nanostructured materials, such as nanopillars: characterized area is generally larger than in other sensors (for example waveguide-based sensors), and the defects or fabrication deviations in a small area of the sensing cell is corrected by averaging the contribution of all the pillars in the cell. The complexity of fabrication processes for single-layer and porous material is lower compared with other sensors because it does not include lithography steps; however, nanostructured materials can be fabricated in large areas using laser interference lithography [39] or nanoimprinting lithography, which can reduce the cost per single device, compared with other techniques such as e-beam lithography.

The possibility of measuring vertically simplifies the optical coupling system, and allows measuring just with a broadband light source which passes through a lens or a microscope objective and a spectrometer. Main disadvantage of this configuration is that the characterized area is larger (the spot diameter could be in the order of 200–300 μm), which reduces the integration capabilities. This area can be reduced, but usually increasing the complexity of the setup [59]. Monitoring the change in the total reflected power instead of the position of the resonance is also an interesting option for reducing complexity and total cost of the sensor [64].

In terms of biofunctionalization processes, the use of vertical sensing cells does not represent great differences compared with other types of sensors; although nanostructured materials are more complex to work with, there is an important increase in the sensing surface and thus in the available bioreceptors to detect. Finally, fluidic dry conditions not only simplifies the fluidic setup but also can increase the sensitivity of a sensing cell, for the higher refractive index contrast between air and bioanalyte, compared with water of PBS and bioanalyte.

Regarding future perspectives, technology based on F-P sensing cells, and characterized measuring total reflected power instead the position of a resonance, and based on IROP concept [64] is nowadays being exploited and commercialized by a biosensing focused company [66], which gives an idea that, when the design of all the components is correctly done, and the same can be said from the integration, which has other particularities that are out of the goals of this work, technology based on vertical sensing cells can be competitive in terms of limit of detection, selectivity, and also in terms of cost compared with other companies in the market. Future developments from the more basic research point of view will include the improvements of the design of the sensing cell (new materials, new configurations), implementing and optimizing fabrication techniques such as NIL for making also competitive cells such as R-NPs arrays and optimized biofunctionalization protocols for a variety of sensing cells and bioanalytes.

## Conclusions

In this work, we review vertically interrogated optical sensors, focusing mainly in the work developed by our group during past years, and the most important aspects that have to be taken into account from the point of view of engineering, in the design of a vertical biosensor. Main advantages of vertical sensors are the lower complexity in light coupling, the lower requirements in fabrication process, and sometimes a simpler fluidic system, in particular when measuring in dry conditions, in comparison with other optical sensors, especially planar sensors.

Four basic aspects of the design are identified and described: design of the sensing cell, fabrication, fluidics and biofunctionalization, and optical characterization techniques. It is clear that all aspects are related, and all of them have to be considered to achieve a reliable device. These four aspects are sensing cell design, fabrication, optical characterization, and fluidics and biofunctionalization processes. Full understanding of these four aspects and their interrelation is a key factor to develop finally a commercial point of care device, which should be nowadays the goal to pursue by people working in biosensors field.
